# Research hotspots and trends in intrinsic capacity of older people in the context of population aging based on CiteSpace

**DOI:** 10.3389/fragi.2026.1640220

**Published:** 2026-03-04

**Authors:** Minhua Ren, Hongtao Guo, Ye Tian, Yingjie Guo, Wanjun Guo, Liangjin Zhu

**Affiliations:** 1 School of Nursing, Inner Mongolia Medical University, Hohhot, China; 2 Nursing Department, The Affiliated Hospital of Inner Mongolia Medical University, Hohhot, China; 3 Department of Neurosurgery, The Affiliated Hospital of Inner Mongolia Medical University, Hohhot, China

**Keywords:** bibliometrics, intrinsic capacity, older people, research hotspot, research trend, visual analysis

## Abstract

**Objective:**

To employ bibliometrics to identify the research hotspots and development trends of the intrinsic capacity of older people, thereby providing novel insights for future studies on the management of intrinsic capacity in older adults and further facilitating the advancement of healthy aging.

**Methods:**

The literature on the intrinsic capacity of older people, published by China National Knowledge Infrastructure, Wanfang, VIP, and SinoMed, as well as the Web of Science Core Collection, was searched from January 2015 to December 2024. The Note Express 4.1.0 software was used for literature management, and the CiteSpace 6.4. R1 software was used to visually analyze the characteristics of the number of publications, countries, institutions, authors, and keywords, and to plot the relevant graphs.

**Results:**

A total of 97 Chinese literature and 745 English literature were included. The number of publications on the older adults’ intrinsic capacity has shown an increasing trend both domestically and internationally. In the past 5 years, intrinsic capacity has received extensive attention, with 96.9% of Chinese papers and 79.6% of English papers published from 2020 to 2024. European researchers collaborate closely, whereas Chinese research shows weaker collaboration and strong regionalism. Keyword analysis identified 6 clusters in Chinese literature and 17 in English. Chinese hotspots mainly include influencing factors, evaluation tools, chronic disease, grip strength, frailty, and falls. Future directions focus on quality of life, social support, and the recovery period. English studies center on association, management, muscle strength, frailty, exercise, and social support, with potential future directions in supplementation and inflammation.

**Conclusion:**

Currently, Chinese research primarily focuses on analyzing influencing factors, with relatively few studies examining intervention measures, while English research has progressed to exploring of intervention strategies. As population aging accelerates, cross-institutional and interdisciplinary cooperation is becoming a key trend. Future research should deepen understanding, particularly for high-risk groups, and explore the effects of supplements, the predictive role of inflammatory markers, and the effectiveness of interventions for this population. These endeavors will contribute to providing precise and personalized management programs for older adults, enhance social support and quality of life, and promote the comprehensive implementation of healthy aging strategies.

## 1 Introduction

According to the 2022 United Nations Population Data Statistics, by 2050, the global population aged 65 and above is projected to constitute 16.4% of the total population, with China’s elderly population reaching approximately 365 million (UN, 2022). As the proportion of elderly individuals grows, demands on the social security system, medical services, and the care infrastructure for older people have surged significantly, imposing a heavier burden on society (Kang et al., 2024). In this context, healthy aging has emerged as an inevitable trend in addressing the challenges of an aging society. In 2015, the World Health Organization (WHO) presented a new vision for healthy aging in the World Report on Aging and Health, defining it as “the process of developing and maintaining the functional ability that enables wellbeing in older age” (World Health Organization, 2015). This functional ability is determined by an individual’s intrinsic capabilities, relevant environmental factors, and their interactions with each other. Notably, Intrinsic Capacity (IC) refers to the composite of all physical and mental capacities of an individual (World Health Organization, 2017). To facilitate the application of IC in clinical settings, the WHO has identified five key domains of IC—movement, vitality, cognition, psychology, and sensation—and introduced an innovative approach, namely, Integrated Care for Older People (ICOPE), which aims to enhance IC and promote healthy aging (Cesari et al., 2022). The decline in IC is a prevalent issue among older adults and is strongly associated with chronic conditions such as cardiovascular disease, chronic kidney disease, and diabetes (Gao et al., 2025; Ramírez-Vélez et al., 2023; Zheng et al., 2025). Research has demonstrated that more than two-thirds of community-dwelling older adults experience IC decline (Cao et al., 2024), whereas older adults with stable or improved IC exhibit significantly lower mortality rates, disability incidence rates, and healthcare utilization rates (Hwang et al., 2024). Early detection of IC decline and timely implementation of targeted interventions are therefore critical not only for enhancing quality of life in later years but also for reducing strain on public health systems. In recent years, research on IC in aging populations has grown rapidly; however, studies exploring its determinants and effective interventions remain in the preliminary stages. Current findings lack systematic synthesis, particularly through bibliometric approaches that enable comprehensive mapping and visual analysis. Therefore, this study aims to systematically and comprehensively review the current research status in the field of IC from 2015 to 2024, and to conduct a bibliometric analysis to accurately identify developmental trends and research hotspots from a macro perspective, thereby providing practical and actionable references for future research.

## 2 Data and methods

### 2.1 Data sources

**FIGURE 1:**
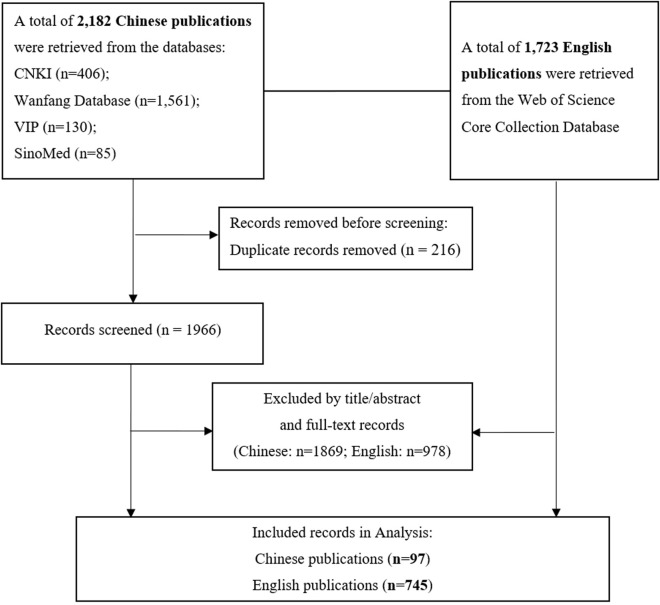
Flow diagram of literature retrieval, screening.

The Chinese literature was sourced from the following databases: China National Knowledge Infrastructure (CNKI), Wanfang Database, China Science and Technology Journal Database (CQVIP), and China Biomedical Literature Service System (SinoMed). In the advanced search function, the subject terms “Intrinsic Capacity of Older Adults” OR “Intrinsic Capacity in Older Adults” were used. All English-language literature was sourced from the Web of Science Core Collection Database. The search formula used was: TS = (“intrinsic capacit*” OR “physical capacit*” OR “mental capacit*” OR ICOPE OR “integrated care for older people”) AND AB = (old* OR elder* OR age* OR senior OR geriatric* OR geronto*) AND Language = English. The search period spanned from January 2015 to December 2024. The source category encompassed all journals, with a particular emphasis on academic journals. A total of 2,182 Chinese records and 1,723 English records were retrieved initially. Subsequently, the selected Chinese literature was imported into Note Express 4.1.0 software for deduplication; then, all the retrieved literature was manually screened by two researchers according to predefined inclusion and exclusion criteria. The retrieval process is shown in Figure 1. Additionally, this bibliometric study did not require ethical approval as it involved no human or animal experimentation.

### 2.2 Inclusion and exclusion criteria

1. Inclusion Criteria: ① Studies focusing on the intrinsic capacities of older adults. ② Literature published in Chinese or English.
2. Exclusion Criteria: ① Duplicate records. ② Non-peer-reviewed materials such as news articles, books, monographs, conference proceedings, newspapers, letters, etc. ③ Documents with incomplete or missing content.

### 2.3 Methods

Descriptive statistical analysis of publications was performed using Microsoft Excel 2019, covering the number of published articles, authorship, co-citations of literature, and keywords. CiteSpace V6.4. R1 was used to visualize co-citations among authors, institutions, countries, keywords, and literature, and to generate a knowledge graph (Chen, 2006). Bibliographic records meeting the inclusion and exclusion criteria were exported to CiteSpace in txt format and renamed as “download_**.txt” for conversion (Chen et al., 2015). The analysis time range was set from January 2015 to December 2024 with a 1-year time slice. Correlation strength was analyzed using the cosine algorithm (g-index, k = 25). Network simplification and key feature highlighting were achieved through Pathfinder, Pruning Sliced Networks, and Pruning the Merged Network. Visualization was performed using Cluster View - Static and Show Merged Network.

## 3 Results

Each node in the figure represents a project, with its size proportional to its occurrence frequency. Larger nodes indicate greater influence within the network. Connections between nodes denote cooperative relationships, where connection thickness reflects the strength of collaboration and color indicates the timing of the first collaboration. The presence of a purple ring around a node signifies high centrality, with wider rings indicating stronger centrality. Nodes with a centrality value of 0.1 or higher are considered key structural elements in the network, serving as critical bridges that connect different researchers or research topics and playing a significant role in facilitating the integration and innovation of domain knowledge (Chen et al., 2015).

### 3.1 Annual publication volume

**FIGURE 2:**
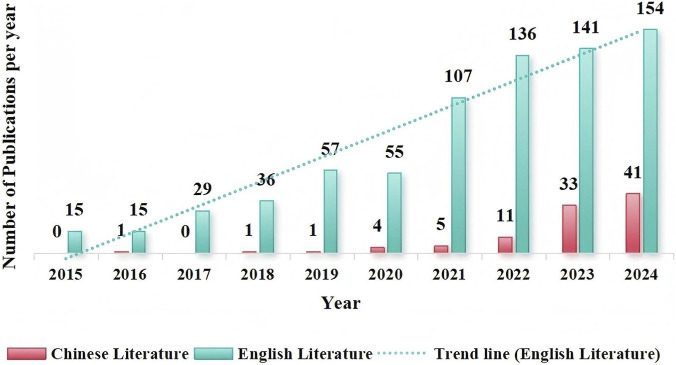
Annual publication volumes on intrinsic capacity in the older people (2015-2024).

**FIGURE 3:**
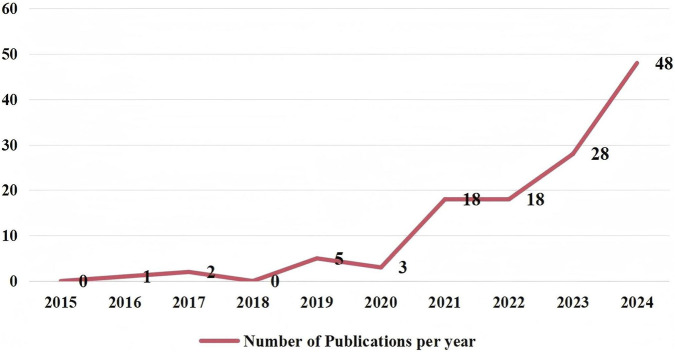
Research trends on intrinsic capacity by Chinese scholars in english journals (2015-2024).

A total of 2,182 studies related to IC in older adults were retrieved from Chinese databases, while 1,732 studies were obtained from English databases. After removing duplicates and applying inclusion and exclusion criteria, 97 Chinese studies and 745 English studies were ultimately selected. The overall number of published articles shows a yearly increasing trend, with the annual number of foreign-language publications generally surpassing that of Chinese publications. The distribution of the annual number of published articles is illustrated in Figure 2. Since 2020, the growth rate of annual publications in both Chinese and English has accelerated. Furthermore, the number of related articles published by Chinese researchers in English journals has also seen rapid growth. The specific number of published papers is detailed in Figure 3.

### 3.2 Country analysis

**FIGURE 4:**
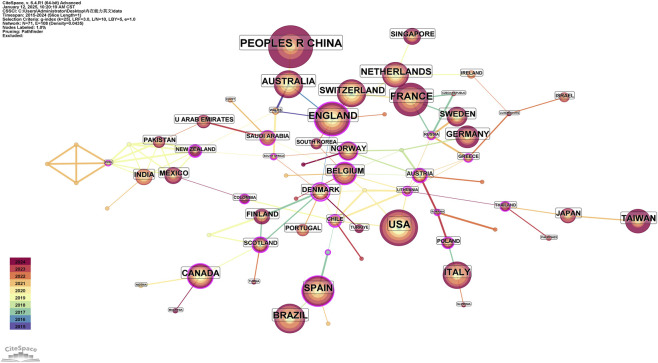
Country co-occurrence network of english literature (2015-2024).

Based on the English literature retrieved from WOS to generate the Country co-occurrence network of IC research for older people (Figure 4). The graph comprises 71 nodes and 108 connections, illustrating the international collaborative network. The top three countries in publication count are China (123), the United States (91), and France (80), while the top three in centrality are Denmark (0.71), Lithuania (0.62), and Chile (0.42).

### 3.3 Institution analysis

**FIGURE 5:**
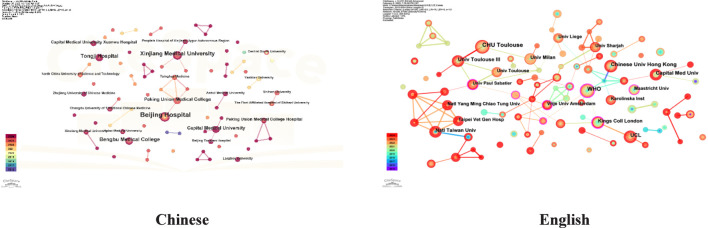
Institutional Co-occurrence network of Chinese and english literature (2015-2024).

The institutional co-occurrence map for research on older adults, based on both Chinese and English literature, is presented in Figure 5. In the Chinese literature, there are 81 nodes and 39 connections, indicating a sparse network of collaboration among institutions. The top three institutions with the highest publication counts are Beijing Hospital (9 publications), Xinjiang Medical University (6), and Bengbu Medical University (5). In contrast, the English literature contains 328 nodes and 397 connections, suggesting stronger collaborative efforts among institutions. The leading institutions in this category are CHU Toulouse (University Hospital of Toulouse, France, 24 publications), the World Health Organization (WHO, 21 publications), and the Chinese University of Hong Kong (20 publications). Notably, the WHO has the highest centrality score of 0.33.

### 3.4 Analysis of authors and co-cited authors

**FIGURE 6:**
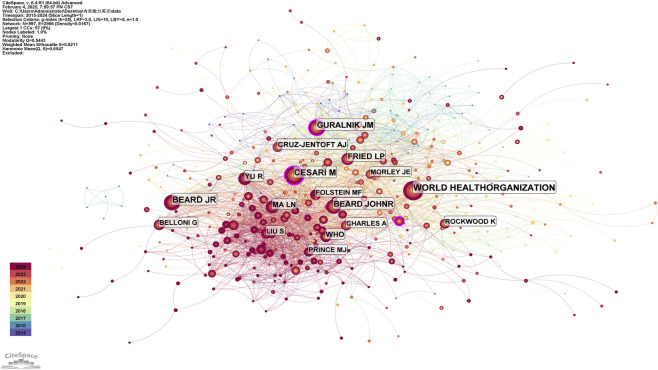
Co-cited map of english literature authors.

Analyzing the author collaboration network in both Chinese and English literature, Table 1 lists the top 10 most prolific authors from the past decade. For the English literature, a co-citation map of authors in the field of research on older individuals with IC has been created (Figure 6). The author with the highest co-citation frequency is the WHO, with a total of 367 citations. The top three authors based on mediating centrality are: CESARI M from France (0.14), GURALNIK JM from the United States (0.13), and DENT E from Australia (0.11). It is noteworthy that the most prolific authors consistently maintain long-standing and stable research teams.

**TABLE 1:** Top 10 authors by publication volume in Chinese and english literature.

Label	Chinese literature			English literature		
	Author (affiliation)	Centrality	Number of publications	Author (country)	Centrality	Number of publications
1	Jie Zhang (Hohai University)	0.02	9	Rolland, Yves (France)	0	17
2	Li Zhang (Anhui medical University)	0	6	Vellas, Bruno (France)	0	16
3	Ji Shen (Beijing hospital)	0	4	Barreto, Philipe de Souto (France)	0.01	13
4	Juan Wu (Beijing hospital)	0.01	4	Beard, John R (United States)	0.02	12
5	Xin Jiang (Xinjiang medical University)	0	4	Cesari, Matteo (Italy)	0.01	12
6	Dandan Zhang (Beijing hospital)	0	3	Gonzalez-bautista, Emmanuel (France)	0	11
7	Shuo Liu (Peking Union medical College hospital)	0	3	Karim, Asima (United Arab Emirates)	0	11
8	Fenghui Chen (Xinjiang medical University)	0	3	Chen, Liang-Kung (Taiwan, China)	0	10
9	Xuan Ma (Xinjiang medical University)	0	3	Qaisar, Rizwan (United Arab Emirates)	0	10
10	Lu Liu (Henan Provincial People’s hospital)	0	3	Ma, Lina (China)	0	10

### 3.5 Co-cited reference analysis

Based on the English literature, the top 10 co-cited references (Beard et al., 2019; Cesari et al., 2018; Beard et al., 2022; Belloni and Cesari, 2019; Charles et al., 2020; Ma et al., 2020; WHO, 2025; Ma et al., 2021; Stolz et al., 2022; González-Bautista et al., 2021) related to IC is presented in Table 2. Co-cited literature forms the foundational knowledge base for this research area. The analysis reveals that these top 10 references can be categorized into four types: five focus on conceptual and theoretical research (Beard et al., 2019; Cesari et al., 2018; Beard et al., 2022; Belloni and Cesari, 2019; WHO, 2025), two examine the validity of screening tools (Ma et al., 2020; González-Bautista et al., 2021), two address the prediction of adverse outcomes (Charles et al., 2020; Stolz et al., 2022), and one represents a cross-sectional study (Ma et al., 2021). Among these, “The structure and predictive value of intrinsic capacity in a longitudinal study of ageing” by Beard et al. (Beard et al., 2019)stands out as the most influential paper, which clarified the predictive role of intrinsic capacity on elderly functional status and proposed several factors with potential research value through long-term follow-up of 2,560 elderly people in England, providing theoretical basis and direction reference for subsequent research.

**TABLE 2:** Top 10 Co-cited references in english literature.

Label	Author	Years	Co-cited reference	Count
1	John R Beard	2019	The structure and predictive value of intrinsic capacity in a longitudinal study of ageing	123
2	Matteo Cesari	2018	Evidence for the domains Supporting the Construct of intrinsic capacity	110
3	John R Beard	2022	Intrinsic capacity: Validation of a new WHO concept for healthy aging in a longitudinal Chinese study	69
4	Giulia Belloni	2019	Frailty and intrinsic capacity: Two distinct but related constructs	62
5	Alexia Charles	2020	Prediction of adverse outcomes in nursing Home Residents according to intrinsic capacity proposed by the world health organization	56
6	Lina Ma	2020	Integrated care for older people screening tool for measuring intrinsic capacity: Preliminary findings from ICOPE Pilot in China	56
7	WHO	2019	Integrated care for older people (icope): guidance for person- centred assessment and pathways in primary care	52
8	Lina Ma	2021	Cross-sectional study examining the status of intrinsic capacity decline in community-dwelling older adults in China: Prevalence, associated factors and implications for clinical care	50
9	Erwin Stolz	2022	Intrinsic capacity predicts negative health outcomes in older adults	49
10	Emmanuel González-Bautista	2021	Screening for intrinsic capacity impairments as markers of increased risk of frailty and disability in the context of integrated care for older people: Secondary analysis of MAPT	45

### 3.6 Keywords analysis

#### 3.6.1 Co-occurrence analysis

**FIGURE 7:**
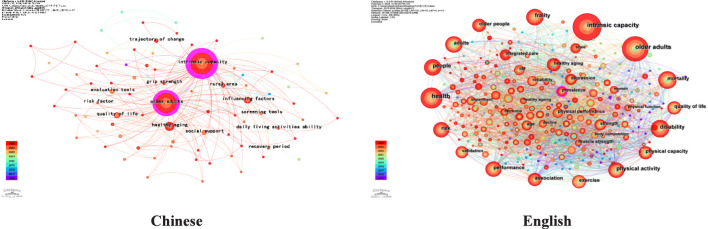
Keyword co-occurrence map of Chinese and english literature (2015-2024).

Based on the Chinese and English literature, a co-occurrence network of keywords in the field of IC was created (Figure 7). In the Chinese literature, six high-frequency keywords (occurring five times or more) were identified. The top three keywords, evaluated by centrality, were “intrinsic capacity (1.17),” “older adults (0.70),” and “healthy aging (0.04)” This analysis concluded that the main research areas in the domestic field of IC for older adults focus on healthy aging, encompassing key aspects such as influencing factors, quality of life, and social support. In contrast, the English literature revealed 139 high-frequency keywords. The top three keywords, according to centrality, were “interventions” (0.41), “activities of daily living” (0.36), and “associations” (0.19).”Other keywords such as “older people,” “health,” “disability,” “frailty,” and “exercise” showed strong co-occurrence and high frequency, suggesting that various research hotspots are closely interconnected. The top 10 high-frequency keywords in both Chinese and English are presented in Table 3.

**TABLE 3:** Top 10 high-frequency keywords in english and Chinese literature.

Label	Chinese literature			English literature		
	Count	Centrality	Keywords	Count	Centrality	Keywords
1	92	1.17	Intrinsic capacity	204	0.1	Older adults
2	74	0.70	Older adults	202	0.01	Intrinsic capacity
3	19	0.04	Healthy aging	156	0.09	Health
4	13	0.01	Influencing factors	108	0.05	Disability
5	7	0.03	Quality of life	102	0.03	Frailty
6	5	0.00	Social support	96	0.07	People
7	5	0.00	Evaluation tools	94	0.18	Physical activity
8	4	0.00	Rural area	87	0.08	Performance
9	4	0.00	Trajectory of change	86	0.04	Adults
10	3	0.03	Risk factor	85	0.01	Mortality

#### 3.6.2 Cluster analysis

**FIGURE 8:**
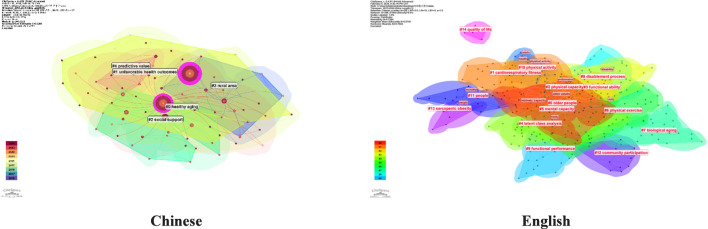
Keyword clustering map of Chinese and english literature (2015-2024).

Based on the keyword co-occurrence network, we conducted a cluster analysis using the log-likelihood ratio (LLR) algorithm. The keyword cluster map is displayed in Figure 8, where the size of each cluster is inversely proportional to its sequence number.

The clustering results of Chinese literature revealed 6 cluster labels, with a modularity Q = 0.3533 (>0.3) and an average silhouette S = 0.7261 (>0.7), indicating a highly reasonable clustering outcome. The three largest clusters by scale are: “#0 Health aging”, “#1 Unfavorable health outcome”, and “#2 Social support”. The clustering results of the English literature indicate that the formation of 17 cluster labels, with a modularity Q = 0.7244(>0.3) and an average silhouette S = 0.8745(>0.7), signifying a relatively robust clustering effect. The three largest clusters by scale are: “#0 Older People”, “#1 Cardiorespiratory fitness”, and “#2 Physical capacity”.

#### 3.6.3 Timeline analysis

**FIGURE 9:**
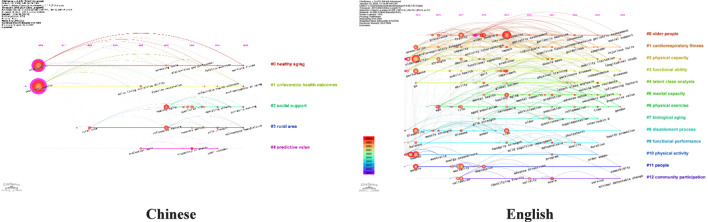
Timeline map of key words in Chinese and english literature (2015-2024).

In comparison to English keywords, Chinese keywords emerged later and showed weaker continuity. Research on IC for the older adults in China began relatively recently and did not reach its peak until 2020. Foreign keywords such as “older adults,” “physical capacity,” and “functional ability” have demonstrated temporal continuity from 2015 to 2024, highlighting these area as primary focuses of IC research for older individuals. The timeline map of key words is presented in Figure 9.

#### 3.6.4 Burst analysis

**FIGURE 10:**
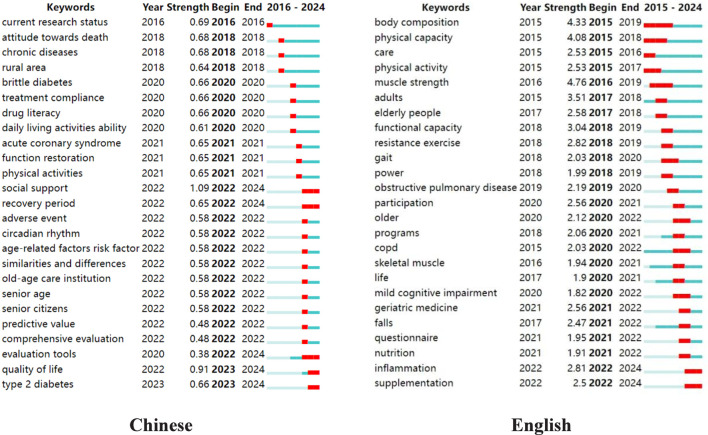
The top 25 keywords with the strongest citation bursts in Chinese and english literature (2015–2024).

Based on the analysis of keyword clustering for burst terms, the top 25 keywords with the strongest citation are illustrated in Figure 10. In this graph, the horizontal axis represents the timeline. The light blue line indicates that a keyword has not yet emerged, the dark blue line marks the beginning of its burst, and the red line highlights the period of peak burst intensity. Among the burst terms in Chinese literature, the top three keywords with the highest burst intensity are “social support” (1.09), “current research status” (0.69), and “chronic disease” (0.68).

Furthermore, the number of burst terms from 2020 to 2024 is significantly higher than those from 2015 to 2019. In English literature, the top three keywords with the greatest burst intensity are “muscle strength” (4.76), “body composition” (4.33), and “physical capacity” (4.08).

## 4 Discussion

### 4.1 Basic information

Research on IC for older adults has seen a consistent year-on-year increase, both domestically and internationally. Studies conducted abroad began earlier than those in China. From the initial introduction of the concept in 2015 until 2018, China published fewer than one paper on average per year. However, following the outbreak of the COVID-19 pandemic in 2019, which severely threatened human safety and health (Pan, 2020), significant challenges arose for implementing healthy aging initiatives. This situation sparked increased interest in researching IC for older adults, drawing considerable attention from scholars worldwide. As a result, the number of published papers has rapidly increased. Notably, among English literature, Chinese scholars lead in the number of published papers and show a relatively fast growth rate, contributing to approximately 16.5% of the total publications, which indicates that China places significant emphasis on addressing the challenges posed by an aging society and maintains sustained strategic support in this domain as a populous nation.

National co-occurrence analysis indicates that China ranks first in English research on older people IC, followed by the United States and France. European countries, such as Denmark, Lithuania, Chile, and Austria, exhibit strong cooperation with one another, while China primarily collaborates with Australia and Japan (Huang et al., 2021; Si et al., 2023). Further co-occurrence analysis of institutions and authors reveals that domestic research teams predominantly engage in cooperation within their own regions rather than seeking cross-regional partnerships. The core group of authors involved in older people’s IC research is mainly concentrated in Beijing, where significant research advancements have been made (Yu et al., 2018), partly due to the establishment of the National Geriatric Medical Center at Beijing Hospital in 2018. In English research, the WHO plays a central role. Among the top ten institutions ranked by publication volume, China accounts for 30% of the total. In contrast, France, Switzerland, the United Kingdom, Sweden, and Milan collectively represent 70%. This indicates that China has an advantage in this field. Therefore, it is recommended that domestic scholars actively enhance collaborations across regions, disciplines, and interdisciplinary teams to promote more in-depth research on older individuals’ issues, facilitating earlier identification and effective interventions.

### 4.2 Research hotspot

#### 4.2.1 Common research hotspots

Through a comprehensive analysis of high-frequency keywords, keywords with high centrality, and cluster results, the investigation of association factors has been identified as a common research focus in both Chinese and English literature. However, there are differences in specific methods between Chinese and English research: Chinese literature often uses current situation survey methods to analyze the influencing factors of specific population IC, while English literature focuses on exploring the correlation between a certain indicator and IC through correlation studies. Current research suggests that older adults’ IC arises from a combination of demographic factors, personal traits, behavioral patterns, and environmental conditions (Ke et al., 2024). Recent studies have particularly emphasized several key areas, including muscle strength, chronic disease, frailty, and falls.

1. The relationship between muscle strength and IC has emerged as a prominent research focus in both domestic and international academic communities. Chinese scholars conducted a survey and analysis of 5,520 elderly individuals using data from the China Health and Retirement Longitudinal Study (CHARLS), which indicates that grip strength is not only a direct measure of muscle strength, but also a predictive indicator of multiple dimensions of IC in older adults beyond cognition (Zhao S. et al., 2024). However, a large-scale meta-analysis incorporating 77 studies demonstrated a significant association between grip strength and cognitive function in older adults (Amini et al., 2024). Therefore, future research needs to further explore and clarify the underlying reasons for these differences.
2. The chronic diseases, such as hypertension, diabetes, depression, and dementia, are closely linked to IC in older adults. A cohort study with a 3.3-year follow-up demonstrated that, among IC domains, only vitality impairment was significantly associated with an increased risk of T2DM (Gao et al., 2025). A study divided 206 older adults with hypertension into a group with decreased IC and a group without such a decline, and conducted 24-h ambulatory blood pressure monitoring. The results revealed a significant association between decreased IC and 24-h systolic blood pressure variability in elderly hypertensive patients (Huang et al., 2022). Two studies analyzed data from 426,714 to 366,406 participants registered in the United Kingdom Biobank, respectively, and found that higher intrinsic capacity impairment scores were significantly associated with an increased risk of both depression and dementia (Ramírez-Vélez et al., 2025; Sun et al., 2024). Therefore, the relationship between different diseases and IC warrants further in-depth investigation. Some diseases affect only a specific dimension of intrinsic capacity, and the current body of related research remains limited, which hinders its application in clinical practice and scientific inquiry. Future studies should strengthen research on the associations between various diseases and the intrinsic capacity to clarify underlying mechanisms and inform the development of targeted intervention strategies.
3. Frailty is closely linked to IC. A decline in IC can precede the clinical manifestations of frailty, which increases the risk of negative outcomes such as frailty, falls, and the inappropriate use of medication (Zhao J. et al., 2024; You et al., 2023; Zhang Y. et al., 2024; Shi et al., 2023). A study involving 665 elderly patients with gastrointestinal cancers found that impaired IC was associated with both frailty and a decreased overall survival rate in these patients (Maheshwari et al., 2024). However, further research is needed to elucidate the complex relationship between these two concepts.
4. Falls interact with IC. Studies have demonstrated that experiencing falls in the past year is a significant factor influencing IC in older adults (Zhang L. et al., 2024), and a decline in IC among older individuals can further heighten their risk of falling (Song et al., 2024). Therefore, healthcare professionals should pay closer attention to elderly patients with reduced IC and a higher risk of falls, implementing targeted interventions to prevent the harmful cycle of decreased IC and increased fall risk.

#### 4.2.2 Distinct research hotspots

##### 4.2.2.1 Chinese research hotspots

Chinese literature tends to focus more on evaluation tools in IC research. This is reflected in high-frequency keywords such as “screening tools,” “evaluation tools,“.

High-quality screening and assessment tools are the foundation for researching IC. Currently, there is no unified measurement standard across different dimensions, and there is a lack of IC assessment tools and comprehensive scoring thresholds that align with various country conditions, medical environments, and older people’s care systems (Yue et al., 2024). Previous studies have predominantly relied on the calculation model of composite scores derived from various assessment tools. The ICOPE tool, proposed by the WHO in 2019 (Takeda et al., 2020), was translated into Chinese by Li Xiaxia et al., in 2024 (Li et al., 2024). This tool facilitates a stepwise process of screening, assessment, plan formulation, implementation, and tracking to maintain the IC of the older people (Takeda et al., 2020). However, its sensitivity and specificity are limited, making it challenging to conduct precise assessments in certain real-world scenarios (Ma et al., 2024). Yan Zhili et al. developed a reliable and valid assessment scale for IC in older adults, focusing on five key dimensions (Yan et al., 2024). After testing, this scale proved effective for evaluating the IC levels of older individuals. However, further large-scale and multi-center validation is necessary to improve its generalizability. Recently, researchers have created an innovative support platform aimed at promoting healthy aging in older adults through IC monitoring (Kolakowski et al., 2025). This platform not only stores health measurement data and offers personalized recommendations but also predicts potential changes in IC over the coming years. It has been successfully implemented in Austria, Italy, and Romania. Therefore, future research should concentrate on developing and validating screening and assessment tools for IC in older adults, aiming for straightforward and accurate identification of IC levels.

##### 4.2.2.2 English research hotspots

English literature primarily focuses on intervention measures in IC research. This is reflected in high-frequency keywords such as “management,” “exercise,” and “social support” as well as high centrality keywords like “interventions” and “activities of daily living” along with cluster analysis results.

Effective intervention measures are essential for alleviating or preventing the decline of IC in older adults. Currently, research on intervention strategies for enhancing IC in this population remains limited, and the formulation of intervention plans requires further refinement. Guo et al. conducted a latent class analysis of elderly individuals in community health service centers, categorizing their IC into three subgroups. They suggested that targeted intervention measures should be implemented for each subgroup to enhance IC effectively (Guo et al., 2024).

1. At present, most intervention programs for older adults’ IC primarily focus on exercise-based interventions. Regular exercise leads to various physiological adaptations and contributes to the improvement and maintenance of vitality and functional status (Jones et al., 2024). A 3-month randomized controlled trial involving 188 elderly individuals demonstrated that a 12-week multi-component exercise program is an effective strategy for enhancing IC, specifically in terms of locomotion, cognition, and vitality capacity among the older adults (Sánchez-Sánchez et al., 2022). A meta-analysis shows that Tai Chi, as a low-cost and low-risk intervention, can significantly enhance IC in older adults, particularly in the domains of cognition, locomotion, and psychological, with consistent effects observed across varying intervention durations and implementation settings (Lan et al., 2025). It can be seen that different exercise forms have distinct effects on various dimensions of IC, and further research in this area should be prioritized in the future.
2. Another key intervention is social support, which enhances psychological resilience, promotes cognitive and functional capacity, and delays functional decline through the provision of emotional support, practical assistance, and informational guidance. A cross-sectional survey of 1,181 older adults in communities showed that subjective support can mitigate the association between lower IC and poorer mental health–related quality of life (Yu et al., 2023). However, a longitudinal study exploring the interaction of IC, social participation, and family support on the trajectory of activities of daily living (ADL) in older adults found that life care helps maintain functional abilities in individuals with intact IC, but may accelerate functional decline in those with low IC. Therefore, future research should further investigate the mechanisms through which social support influences the IC to identify appropriate intervention approaches and optimal levels.

### 4.3 Research trends

#### 4.3.1 Chinese research trends

Based on the burst terms identified in Chinese literature, the social support, quality of life, evaluation tools, recovery period, and type 2 diabetes may represent potential areas for future research development on IC in older adults. Since the evaluation tools, social support, and type 2 diabetes have been mentioned, this section will only elaborate on the recovery period and quality of life.

The quality of life is a critical indicator for evaluating healthy aging. Enhancing the quality of life is a prerequisite for achieving the goal of a “secure and enjoyable old age.” Research has demonstrated that impaired IC not only significantly impacts the quality of life for older adults but also negatively affects their perception and use of social support (Zhao et al., 2023; Jiang et al., 2023). A comprehensive social support system can provide older individuals with positive emotional experiences, which can strengthen their sense of belonging and wellbeing. Therefore, future research in China may focus on the development of a social support system to enhance the quality of life among older adults.

Elderly patients during the rehabilitation period frequently experience delayed recovery of physical and immune functions, are prone to psychological anxiety, and often have comorbid chronic diseases. Furthermore, those with impaired IC face a significantly higher risk of delayed or inadequate postoperative recovery, necessitating a longer and more intensive rehabilitation process. Currently, research on disease recovery and IC remains relatively limited, with only a few studies focusing on stroke and postoperative patient populations. Among older adults recovering from stroke, IC is generally low and positively correlated with self-efficacy (Li et al., 2022). A longitudinal study demonstrated that, among elderly cancer patients, frailty is a stronger predictor of postoperative functional recovery and quality of life than IC (Hu et al., 2025). However, evidence also indicates that older adults with diminished IC are more likely to develop frailty. Therefore, researchers should investigate the underlying mechanisms linking frailty and IC and clarify their predictive value for functional outcomes during the recovery period.

#### 4.3.2 English research trends

The burst analysis results of English literature indicate that supplements and inflammation are likely to become key research directions in the future.

It remains unclear whether nutritional supplements can help maintain the balance of energy and nutrition in older individuals, thereby improving their IC. To date, no relevant studies have been identified in China. A RCT carried out by French researchers among community-dwelling elderly participants, who received either ω-3 supplements or a placebo as part of a multi-domain intervention, demonstrated no statistically significant difference in IC between the control group and the experimental group (Giudici et al., 2020). Therefore, further in-depth experiments and discussion are still needed in this field.

Identifying inflammatory markers that are closely associated with IC is essential for developing strategies to prevent or slow age-related functional decline and dependence on care. A study investigated the relationship between blood biomarkers related to inflammation and neurodegeneration and IC. The findings indicated that interleukin-6 is associated with a rapid decline in IC in men (de Assunção Cortez Corrêa et al., 2025). Additionally, a 5-year follow-up multi-domain intervention study revealed that both low-grade inflammation and elevated homocysteine levels were associated with impaired overall IC levels in older individuals (Giudici et al., 2019).

In conclusion, future research on IC in older adults should prioritize enhancing social support and quality of life, especially for those with various chronic diseases. It is crucial to elucidate further the correlation between different supplements and IC in this age group, as well as the predictive role of inflammatory markers on IC, to maximize the potential for primary prevention of IC.

## 5 Conclusion

With the continuous acceleration of population aging, elderly healthcare has become a core issue in achieving healthy aging. A growing number of scholars are now focusing on research into IC in older people, particularly in European countries where the aging issue emerged earlier. China, being a highly populated country, is also facing a serious aging problem. In recent years, there has been significant progress in research related to IC in older people, with a notable increase in the number of published papers on this topic. Currently, research on IC in older adults is focusing on several key areas, which include the development of assessment and screening tools, the identification of influencing factors, and the exploration of effective intervention strategies. In the future, research in this field will place greater emphasis on enhancing social support and quality of life for older adults, improving functional capacity in patients during the recovery period of chronic diseases, evaluating the effectiveness of various supplements, and investigating the predictive role of inflammatory markers on IC. Efforts will also aim to strengthen early detection of signals indicating declines in IC among elderly individuals, along with actively implementing intervention measures to promote healthy aging.

## 6 Strengths and limitations

Our study utilizes the visualization analysis software CiteSpace to perform an in-depth analysis of relevant literature over the 10 years since the concept of Intrinsic Capacity was proposed. This approach enables us to precisely identify research trends and hotspots from a macro perspective, thereby providing practical and actionable references for future research endeavors. However, it is crucial to recognize the limitations of this study. First, the literature search was confined to English and Chinese sources, which might introduce publication bias. Second, the analysis of English literature relied exclusively on the WOS database, possibly resulting in deviations in the research findings. Additionally, recent high-quality articles may not have garnered adequate attention owing to their short publication time frame and low citation frequency, requiring a need for updates in future research.
