# Supplementary material for: Research hotspots and trends in intrinsic capacity of older people in the context of population aging based on CiteSpace
Source: Front Aging. 2026 Mar 4;7:1640220. doi: 10.3389/fragi.2026.1640220 (PMC12996128; doi:10.3389/fragi.2026.1640220)
Supplement: Supplementary file 1 [file Supplementaryfile1.docx]

Appendix [A](https://pmc.ncbi.nlm.nih.gov/articles/PMC11730783/#MOESM1): Search strategy

**Table 1 Search strategy for Web of Science**

| #5 | #4 AND #3 AND #2 AND #1 | 1,723 |
| --- | --- | --- |
| #4 | DOP = (2015-01-01/2024-12-31) | ＿＿ |
| #3 | LA=(English) | 22,854,952 |
| #2 | (AB = (old* OR elder* OR age* OR senior OR senile OR geriatric* OR geronto*)) | 2,640,025 |
| #1 | (TS = ("intrinsic capacit*" OR "physical capacit*" OR "mental capacit*" OR ICOPE OR "integrated care for older people")) | 3,367 |

**Table 2 Search strategy for China National Knowledge Infrastucture (CNKI)**

| #1 | （主题：老年内在能力）AND（时间范围：2015-01-01~2024.12.31）  Translate: (Subject: Intrinsic capacity in older adults) AND (Time horizon: 2015-01-01~2024.12.31) | 367 |
| --- | --- | --- |
| #2 | （主题：老年人内在能力）AND（时间范围：2015-01-01~2024.12.31）  Translate: (Subject: Intrinsic capacity in older adults) AND (Time horizon: 2015-01-01~22024.12.31) | 151 |
| #3 | #1 OR #2 | 406 |

**Table 3 Search strategy for Wanfang Database**

| #1 | （主题：老人）OR（主题：老年）OR（主题：老年人）  Translate: (Subject: old man) OR (Subject: senium) OR (Subject: old people) | 616,567 |
| --- | --- | --- |
| #2 | （主题：内在能力）  Translate: (Subject: Intrinsic capacity) | 67,936 |
| #3 | 时间范围：（2015-01-01~2024.12.31）  Translate: (Time horizon: 2015-01-01~2024.12.31) | ＿＿ |
| #4 | #1 AND #2 AND #3 | 1,561 |

**Table 4 Search strategy for China Science and Technology Journal Database (CQVIP)**

| #1 | (M=(老年 OR 老年人) OR M=老人)  Translate: (M = (old man OR senium) OR (M=old people) | 309,825 |
| --- | --- | --- |
| #2 | (M=内在能力）  Translate: (M=Intrinsic capacity) | 263 |
| #3 | 时间范围：（2015-01-01~2024.12.31）  Translate: (Time horizon: 2015-01-01~2024.12.31) | ＿＿ |
| #3 | #1 AND #2 AND #3 | 130 |

**Table 5 Search strategy for Chinese Biomedical Literature Service System (SinoMed)**

| #1 | "老人"[常用字段:智能] OR "老年人"[常用字段:智能] OR "老年"[常用字段:智能]  Translate: "old man"[Common field: Intelligence] OR "senium"[Common field: Intelligence] OR "old people"[Common field: Intelligence] | 239,209 |
| --- | --- | --- |
| #2 | "内在能力"[常用字段:智能]  Translate: "Intrinsic capacity"[Common field: Intelligence] | 151 |
| #3 | 时间范围：（2015-01-01~2024.12.31）  Translate: (Time horizon: 2015-01-01~2024.12.31) | ＿＿ |
| #4 | #1 AND #2 AND #3 | 85 |
